# Incidence of post-procedural atrial fibrillation after multivessel percutaneous coronary intervention versus coronary artery bypass grafting: a nationwide observational study

**DOI:** 10.1136/openhrt-2026-004030

**Published:** 2026-07-13

**Authors:** Erik Sunnefeldt, Sacharias von Koch, Moman Aladdin Mohammad, David Erlinge, Ole Frobert, Anna Björkenheim

**Affiliations:** 1 Department of Cardiology, Örebro University Faculty of Medicine and Health, Örebro, Sweden; 2 Department of Cardiology, Örebro University Hospital, Örebro, Sweden; 3 Department of Clinical Sciences Lund, Division of Cardiology, Lund University, Lund, Sweden; 4 Department of Cardiology, Skåne University Hospital Lund, Lund, Sweden

**Keywords:** ANTICOAGULATION, Atrial Fibrillation, Coronary Artery Bypass, Percutaneous Coronary Intervention

## Abstract

**Background:**

Postoperative atrial fibrillation (AF) is a common complication after coronary artery bypass grafting (CABG) but is less well characterised after multivessel percutaneous coronary intervention (MV-PCI). With the increasing use of MV-PCI, we compared the incidence and clinical implications of clinically significant post-procedural AF following MV-PCI versus CABG.

**Methods:**

In this nationwide observational study, we analysed data from the Swedish Coronary Angiography and Angioplasty Registry and the Swedish Prescribed Drug Register. We included all patients in Sweden with chronic coronary syndrome who underwent MV-PCI or isolated CABG between August 2013 and October 2020, excluding those with prior AF or oral anticoagulant (OAC) use in the preceding year. Clinically significant post-procedural AF was defined using a treatment-based proxy: a new OAC dispensation within 3 months after the procedure, with persistence at 9 months. The primary outcome was post-procedural AF incidence at 90 days; secondary outcomes were stroke, myocardial infarction, major bleeding and all-cause mortality.

**Results:**

A total of 4471 patients undergoing MV-PCI and 2232 undergoing CABG were included. Post-procedural AF occurred in 0.6% after MV-PCI and 5.7% after CABG (adjusted risk ratio 15.02; 95% CI 8.72 to 25.87; p<0.001). MV-PCI patients were older and had more comorbidities, whereas CABG patients had higher rates of diabetes and more extensive coronary disease. At 90 days, all-cause mortality and major bleeding did not differ significantly. Myocardial infarction was more common after MV-PCI (1.1 vs 0.4%), whereas stroke was more frequent after CABG (0.6 vs 0.3%).

**Conclusions:**

Clinically significant post-procedural AF, defined by initiation and persistence of OAC therapy, was nearly tenfold more common after CABG than after MV-PCI. This association remained robust after multivariable adjustment for demographics, comorbidities and angiographic/severity-related factors. Differences in clinical outcomes were modest and should be interpreted cautiously.

**WHAT IS ALREADY KNOWN ON THIS TOPIC**

- Postoperative atrial fibrillation (AF) is a frequent complication after coronary artery bypass grafting (CABG) and is associated with an increased risk of stroke and adverse outcomes.
- The incidence and clinical implications of post-procedural AF after multivessel percutaneous coronary intervention (MV-PCI) are less well characterised in real-world populations.

**WHAT THE STUDY ADDS**

- Clinically significant post-procedural AF, defined by oral anticoagulant initiation and persistence, was nearly tenfold more common after CABG than after MV-PCI.
- Clinically significant post-procedural AF may be considered in future composite endpoints in comparative revascularisation studies, but requires careful definition and validation.

**HOW THIS STUDY MIGHT AFFECT RESEARCH, PRACTICE OR POLICY**

- These findings highlight procedure-specific AF risks between CABG and MV-PCI and may inform future study design and clinical follow-up strategies.

## Introduction

 Revascularisation with coronary artery bypass grafting (CABG) is associated with a high risk of postoperative atrial fibrillation (AF), which has been linked to adverse outcomes including stroke and all-cause mortality.^1^,^5^

In recent years, multivessel percutaneous coronary intervention (MV-PCI) has been increasingly adopted as an alternative to CABG for selected patients with multivessel coronary artery disease,^6^ facilitated by advances in stent technology and intravascular imaging and by the ability to complete staged multivessel treatment during a single hospitalisation.^7^,^9^ This approach offers shorter hospital stays, faster recovery, and comparable long-term outcomes in selected patients.^10 11^ Yet, compared with single-vessel PCI, MV-PCI often entails longer procedures, greater myocardial stress, and more pronounced haemodynamic fluctuations, factors that may predispose patients to post-procedural AF. However, the incidence of post-procedural AF after MV-PCI remains poorly characterised.

Available data are limited; most studies rely on in-hospital monitoring of patients with ST-segment elevation myocardial infarction, report post-procedural AF rates of 4.5–7% and link these episodes to adverse cardiovascular outcomes.^12^,^16^ In a post hoc analysis of the randomised EXCEL trial comparing PCI with CABG, post-revascularisation AF independently predicted stroke and long-term mortality; however, the PCI-related AF incidence (0.1%) was likely underestimated due to limited detection protocols and inconsistent identification of pre-procedural AF.^17^ Moreover, prior studies seldom distinguish transient, clinically insignificant episodes from sustained AF that require long-term management.

We therefore conducted a nationwide, register-based observational study to compare the incidence and clinical implications of clinically significant post-procedural AF, defined by a new oral anticoagulant (OAC) dispensation after the index procedure, among patients undergoing MV-PCI versus CABG.

## Methods

### Study design and data sources

This nationwide, retrospective, observational study used data from the Swedish Coronary Angiography and Angioplasty Registry (SCAAR) and the Swedish Prescribed Drug Register to identify all patients in Sweden undergoing MV-PCI or isolated CABG for chronic coronary syndrome. SCAAR contains comprehensive information on all angiography and PCI procedures in Sweden, including patient characteristics (eg, age, sex, risk factors), angiographic findings (eg, disease extent, lesion complexity) and treatment strategies (eg, stent implantation, CABG referral). Because SCAAR is continuously updated with vital status data from the Swedish Population Register, and each resident has a unique personal identification number, completeness is high.^18^ We linked SCAAR records with dispensation data from the Swedish Prescribed Drug Register, which captures all dispensed prescriptions in Sweden, to ascertain OAC use.

### Study population

SCAAR was used to identify all patients in Sweden who underwent isolated CABG (without concomitant valve or other cardiac surgery) or MV-PCI during the study period (2013–2020). MV-PCI was defined as implantation of drug-eluting stents in ≥2 epicardial coronary vessels (left anterior descending, right coronary, left circumflex or left main) or their major branches. Exclusion criteria included documented AF or atrial flutter before the index procedure; any OAC dispensation in the year before the index procedure (Anatomical Therapeutic Chemical codes B01AF01, B01AF02, B01AF03, B01AE07, B01AA03); bare-metal stent implantation; unknown procedure time; CHA_2_DS_2_-VASc score ≤1; and revascularisation for indications other than chronic coronary syndrome.

### Outcomes

The primary outcome was clinically significant post-procedural AF, defined as a treatment-based proxy: a new OAC dispensation within 3 months after MV-PCI or CABG, with continued dispensed prescription at 9 months (operationalised as an additional dispensation between 270 and 450 days), to distinguish sustained treatment from short-term OAC therapy for other indications. Because all patients in the cohort had CHA_2_DS_2_-VASc scores ≥2, they would generally be eligible for anticoagulation if AF occurred; thus, a new OAC dispensation was a pragmatic proxy for clinically actionable AF. Secondary outcomes assessed at 90 days post-procedure included ischaemic stroke, myocardial infarction, bleeding events and all-cause mortality. Detailed definitions are provided in online supplemental table S1.

### Statistical analysis

The primary outcome was evaluated within 90 days using Kaplan-Meier survival analysis and adjusted Poisson regression to compare post-procedural AF incidence between revascularisation strategies, yielding risk ratios with 95% CIs. Secondary outcomes were assessed with Kaplan-Meier survival analysis and multivariable Cox proportional hazards models, summarised by HRs with 95% CIs and log-rank p values. Both Cox and Poisson models were adjusted for procedure year, age, sex, diabetes mellitus, hypertension, hyperlipidaemia, prior bleeding, heart failure, chronic kidney disease, angiographic findings, smoking status, Canadian Cardiovascular Society score and previous myocardial infarction, stroke, PCI and CABG. Proportional hazards assumptions were verified using log–log plots and formal statistical tests. In supplementary analyses, we evaluated 1-year secondary outcomes using Kaplan-Meier estimates and multivariable Cox models with the same adjustment strategy as for the 90-day analyses. A sensitivity analysis investigated associations between MV-PCI procedural factors (number of stented vessels, fluoroscopy time, contrast volume) and post-procedural AF risk. All analyses were performed using Stata V.18.0 (StataCorp, College Station, Texas, USA, 2023).

## Results

### Study population characteristics

**Figure 1:**
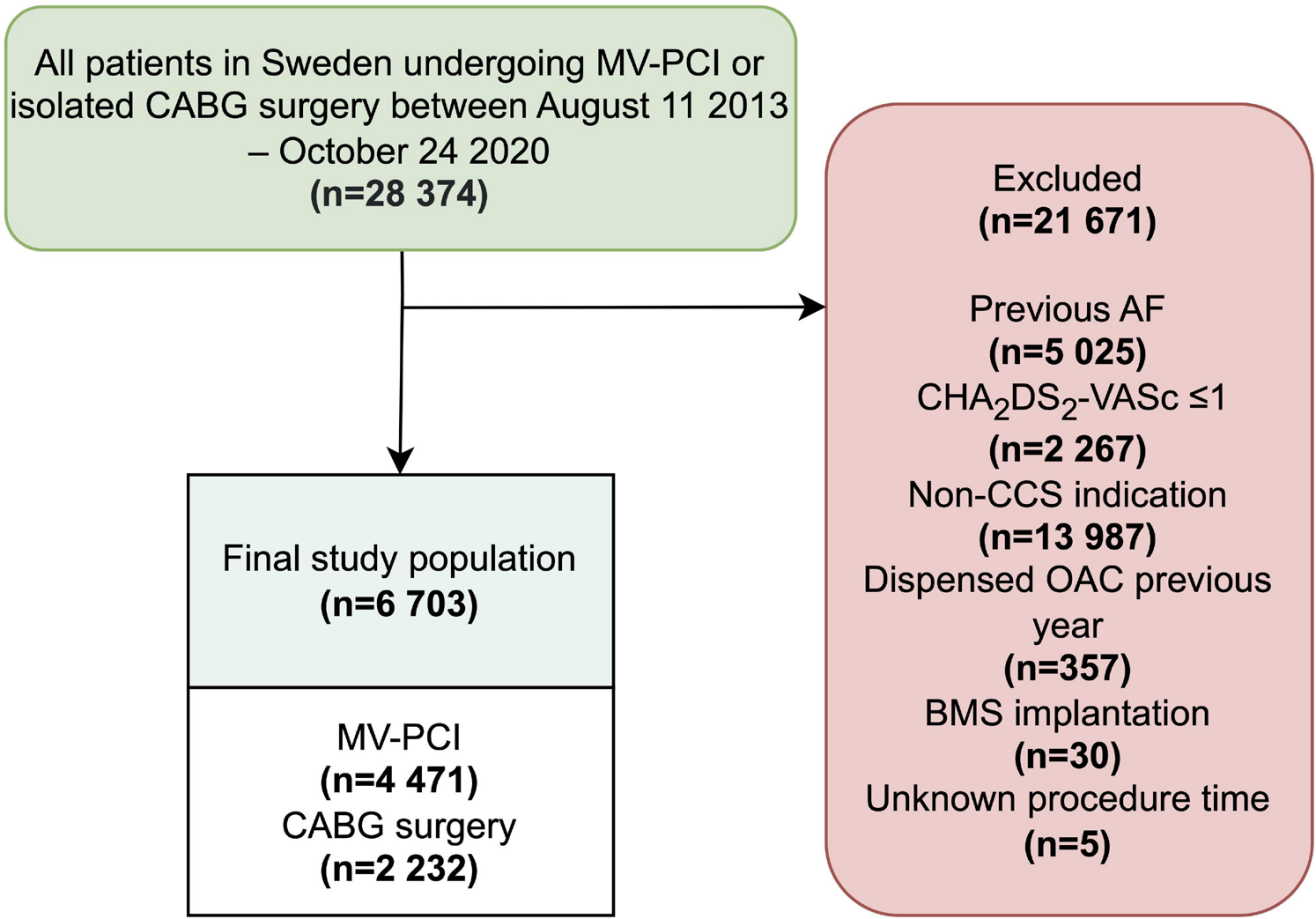
Flowchart of the study population. Schematic representation of patient inclusion (MV-PCI or CABG), application of exclusion criteria and the final sample size. Missing baseline covariates were handled using a complete-case approach and are not shown in the flowchart. AF, atrial fibrillation; BMS, bare-metal stent; CABG, coronary artery bypass grafting; CCS, chronic coronary syndrome; OAC, oral anticoagulant; MV, multivessel; PCI, percutaneous coronary intervention.

We identified all patients in Sweden who underwent MV-PCI or isolated CABG between 11 August 2013 and 24 October 2020 (n=28 374). After applying predefined exclusion criteria, 21 671 patients were excluded for the following reasons: prior AF (n=5025), CHA₂DS₂-VASc score ≤1 (n=2267), non-chronic coronary syndrome indication (n=13 987), OAC dispensation in the previous year (n=357), bare-metal stent implantation (n=30) and unknown procedure time (n=5). The final study cohort comprised 4471 MV-PCI patients and 2232 CABG patients (figure 1).

Patients treated with MV-PCI were older and had higher rates of angina, heart failure, chronic kidney disease and higher CHA_₂_DS_₂_-VASc scores, whereas CABG patients had higher rates of diabetes mellitus and more extensive coronary artery disease. Baseline characteristics are summarised in table 1. The proportion of missing data across baseline covariates was low (8.1%), and a complete-case analysis was used.

**Table 1:** Baseline characteristics

	MV-PCI	CABG	P value
	n=4471	n=2232	
Inclusion period, years			
2013–2015	1524 (34.1%)	869 (38.9%)	<0.001
2016–2018	1850 (41.4%)	833 (37.3%)	
2019–2020	1097 (24.5%)	530 (23.7%)	
Age, years, mean (SD)	68.8 (9.4)	67.0 (8.0)	<0.001
Age ≥75 years, n (%)	1265 (28.3)	379 (17.0)	<0.001
Sex, n (%)			
Male	3503 (78.3)	1871 (83.6)	<0.001
Female	968 (21.7)	361 (16.2)	
BMI, kg/m^2^, mean (SD)	27.5 (4.2)	27.8 (4.0)	0.003
Canadian Cardiovascular Society score grading, n (%)			
I	428 (9.6)	214 (9.6)	0.004
II	2265 (50.8)	1217 (54.7)	
III	1708 (38.3)	778 (35.1)	
IV	61 (1.4)	17 (0.8)	
Smoking status, n (%)			
Non-smoker	1887 (43.5)	909 (41.2)	0.13
Previous smoker	2266 (47.6)	1074 (48.7)	
Active smoker	388 (8.9)	222 (10.1)	
Diabetes mellitus type 2, n (%)	1288 (28.8)	738 (33.1)	<0.001
Hypertension, n (%)	3946 (88.3)	1868 (83.7)	<0.001
Hyperlipidaemia, n (%)	3776 (84.6)	1814 (81.7)	0.002
Previous myocardial infarction, n (%)	1694 (37.9)	509 (22.8)	<0.001
Previous PCI, n (%)	1809 (40.5)	505 (22.7)	<0.001
Previous CABG, n (%)	523 (11.7)	19 (0.9)	<0.001
Heart failure, n (%)	324 (7.2)	76 (3.4)	<0.001
Previous stroke, n (%)	264 (5.9)	104 (4.7)	0.035
Chronic kidney disease, n (%)	162 (3.6)	31 (1.4)	<0.001
Previous major bleeding event, n (%)	188 (4.2)	80 (3.6)	0.22
CHA_2_DS_2_-VASc score, n (%)			
2	967 (21.6)	567 (25.4)	<0.001
3	1346 (30.1)	807 (36.2)	
4	1216 (27.2)	587 (26.3)	
5	656 (14.7)	196 (8.8)	
6	206 (4.6)	61 (2.7)	
7	61 (1.4)	11 (0.5)	
8	18 (0.4)	3 (0.1)	
9	1 (<0.1)	0 (0.0)	
Angiographic findings, n (%)			
2-VD	2171 (51.4)	266 (12.6)	<0.001
3-VD	1286 (30.5)	1072 (50.7)	
1-VD and LM	186 (4.4)	88 (4.2)	
2-VD and LM	288 (6.8)	243 (11.5)	
3-VD and LM	290 (6.9)	447 (21.1)	
Number of stented vessels, n (%)			
Two vessels	3016 (67.4)		
Three vessels	281 (6.3)		
One vessel and LM	839 (18.8)		
Two vessels and LM	301 (6.7)		
Three vessels and LM	34 (0.8)		
Time to CABG, days, median (IQR)		29.0 (18.0–43.0)	
Contrast volume, mL, mean (SD)	219.2 (93.6)		
Fluoroscopy time, min, median (IQR)	22.2 (14.9–34.0)		

Baseline characteristics of patients undergoing MV-PCI versus CABG.

BMI, body mass index; CABG, coronary artery bypass grafting; LM, left main; MV-PCI, multivessel percutaneous coronary intervention; PCI, percutaneous coronary intervention; VD, vessel disease.

### Primary outcome

**Figure 2:**
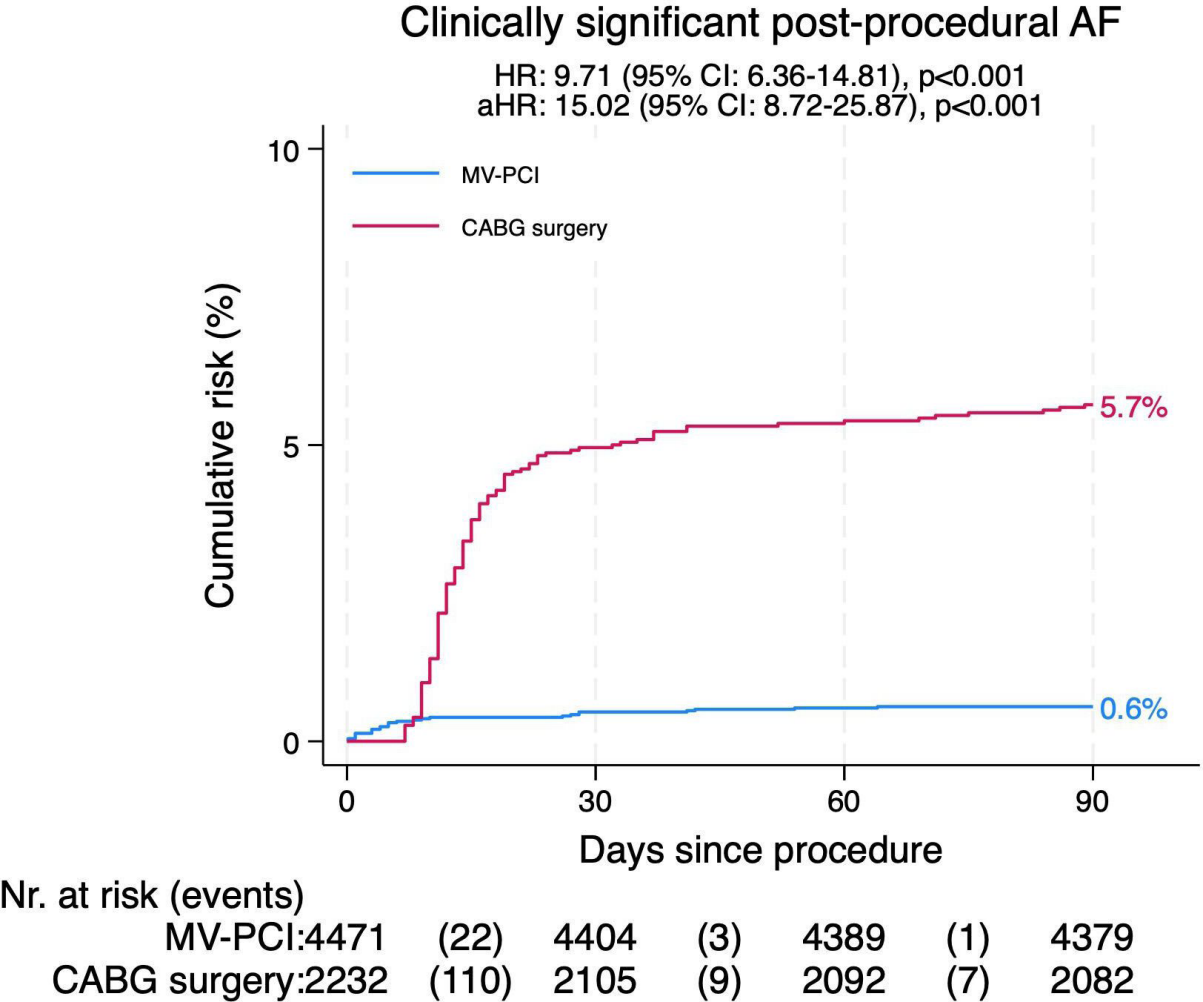
Primary outcome: MV-PCI versus CABG. Kaplan-Meier curves illustrating the 90-day cumulative incidence of clinically significant post-procedural AF, defined as a newly dispensed OAC prescription within 90 days after revascularisation, confirmed by a subsequent dispensation between 270 and 450 days after the index procedure. Time from the index procedure to the first OAC dispensation is shown among patients fulfilling both criteria, stratified by MV-PCI and CABG. The outcome was analysed using Poisson regression in univariable and multivariable models, adjusted for inclusion year, age, sex, diabetes mellitus, hypertension, hyperlipidaemia, prior major bleeding, heart failure, chronic kidney disease, angiographic findings, smoking status, Canadian Cardiovascular Society score and history of myocardial infarction, stroke, PCI and CABG. AF, atrial fibrillation; aHR, adjusted HR; aRR, adjusted risk ratio; CABG, coronary artery bypass grafting; MV-PCI, multivessel percutaneous coronary intervention; OAC, oral anticoagulant; RR, risk ratio.

The incidence of clinically significant post-procedural AF was 0.6% in MV-PCI patients versus 5.7% in CABG patients, corresponding to an adjusted risk ratio for CABG versus MV-PCI of 15.02 (95% CI 8.72 to 25.87; p<0.001) (figure 2).

### Secondary outcomes

**Figure 3:**
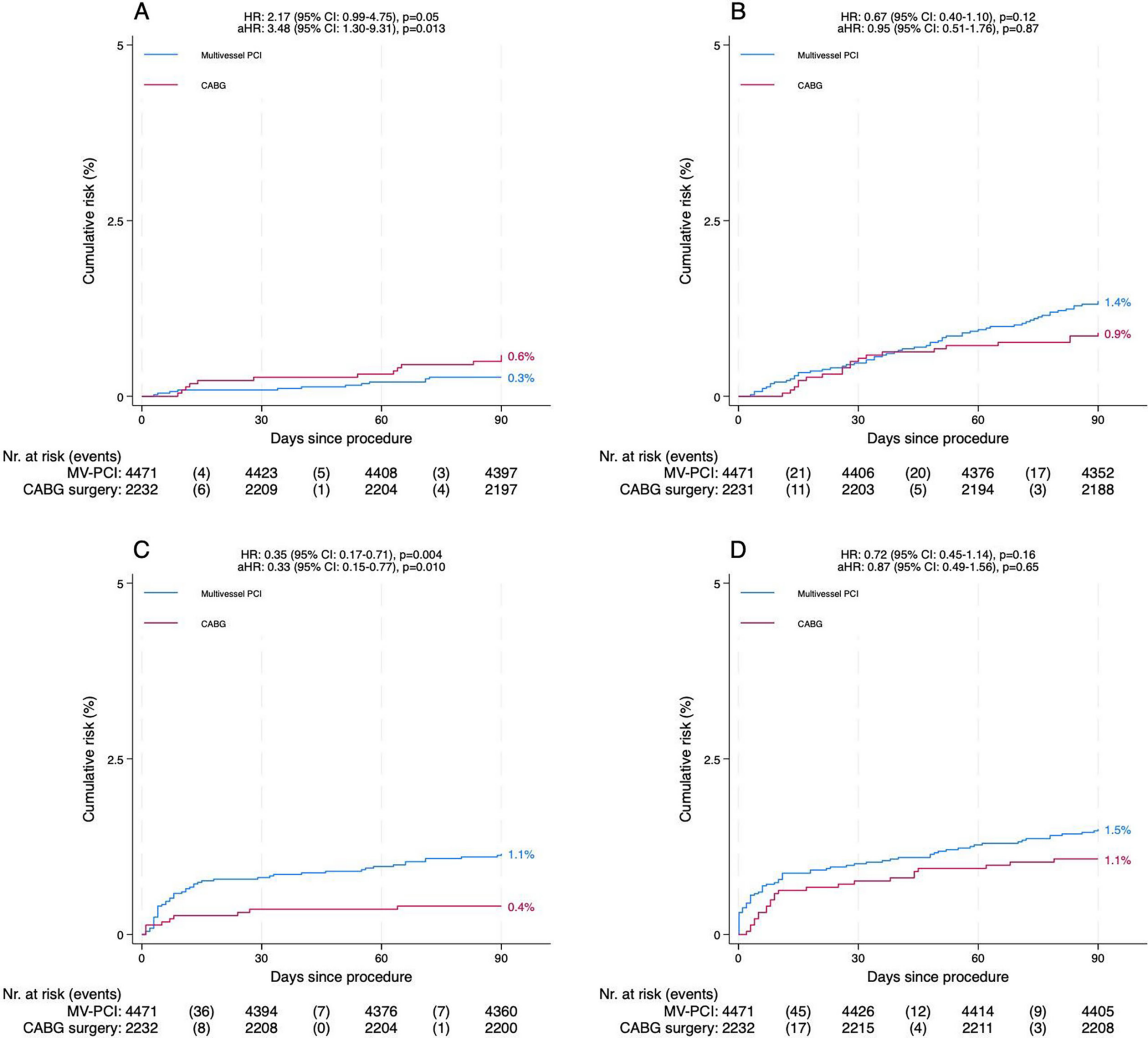
Secondary outcomes: MV-PCI versus CABG. Kaplan-Meier curves illustrating the 90-day event rates of (**A**) stroke, (**B**) bleeding events, (**C**) myocardial infarction and (**D**) all-cause mortality after MV-PCI and CABG. Outcomes were analysed with Kaplan-Meier estimates and both univariable and multivariable Cox regression. The multivariable model was adjusted for inclusion year, age, sex, diabetes mellitus, hypertension, hyperlipidaemia, prior major bleeding, heart failure, chronic kidney disease, angiographic findings, smoking status, Canadian Cardiovascular Society score and history of myocardial infarction, stroke, PCI and CABG. aHR, adjusted HR; CABG, coronary artery bypass grafting; MV-PCI, multivessel percutaneous coronary intervention; OAC, oral anticoagulant.

At 90 days, no significant differences were observed between MV-PCI and CABG patients in all-cause mortality or major bleeding events. Myocardial infarction occurred more often after MV-PCI (1.1% vs 0.4%); the adjusted HR was 0.33 for CABG versus MV-PCI (95% CI 0.15 to 0.77; p=0.010). Stroke was more frequent after CABG (0.6% vs 0.3%); the adjusted HR was 3.48 for CABG versus MV-PCI (95% CI 1.30 to 9.31; p=0.013) (figure 3).

### Subgroup analyses

**Figure 4:**
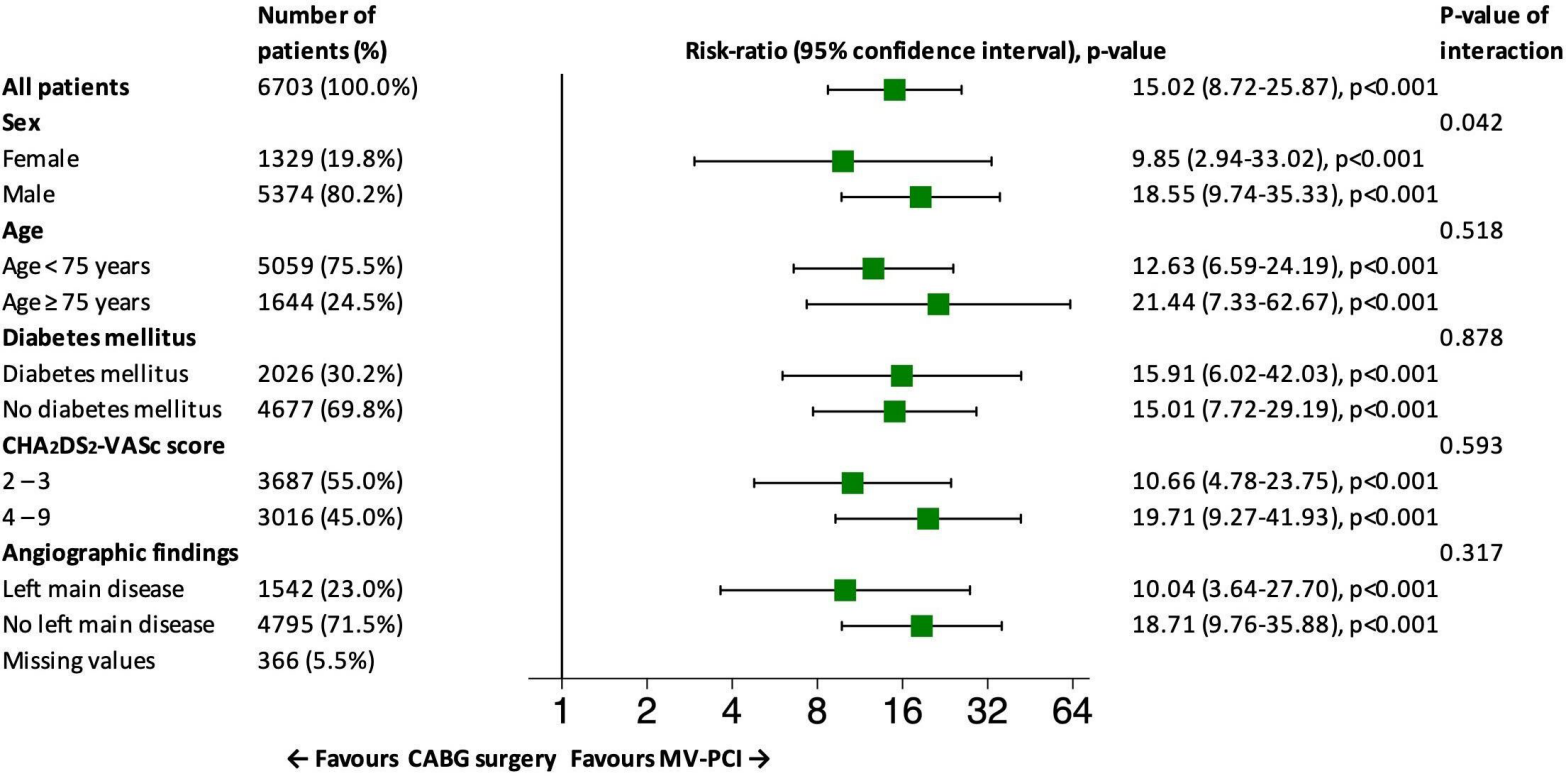
Subgroup analysis of post-procedural atrial fibrillation incidence. Forest plot illustrating adjusted risk ratios for post-procedural atrial fibrillation across predefined subgroups (sex, age, diabetes mellitus, CHA_₂_DS_₂_-VASc score and angiographic extent of disease) with p values for interaction. All models were adjusted for inclusion year, age, sex, diabetes mellitus, hypertension, hyperlipidaemia, prior major bleeding, heart failure, chronic kidney disease, angiographic findings, smoking status, Canadian Cardiovascular Society score and history of myocardial infarction, stroke, PCI and CABG. CABG, coronary artery bypass grafting; MV-PCI, multivessel percutaneous coronary intervention.

The association between revascularisation strategy and post-procedural AF incidence was consistent across predefined subgroups. An interaction by sex (p=0.042) was observed. Detailed results are presented in figure 4.

### Sensitivity and supplementary analyses

In a sensitivity analysis among MV-PCI patients (online supplemental figure S1), relative to two-vessel PCI, three-vessel PCI (adjusted risk ratio 5.12, 95% CI 0.81 to 32.48, p=0.08) and any left main artery involvement (not mutually exclusive with vessel-count categories) (adjusted risk ratio 3.64, 95% CI 0.91 to 14.57, p=0.07) were associated with numerically higher post-procedural AF incidence, although not reaching statistical significance. No significant associations were observed for fluoroscopy time (adjusted risk ratio 2.12, 95% CI 0.81 to 5.51, p=0.12) or contrast volume (adjusted risk ratio 1.24, 95% CI 0.51 to 3.04, p=0.63).

In supplementary analyses assessing clinical outcomes at 1 year, MV-PCI was associated with a higher incidence of myocardial infarction (3.0% vs 0.9%; adjusted HR 0.34 for CABG vs MV-PCI, 95% CI 0.20 to 0.57; p<0.001) and bleeding (3.3% vs 1.7%; adjusted HR 0.63 for CABG vs MV-PCI, 95% CI 0.41 to 0.97; p=0.035). Conversely, CABG was associated with a significantly higher incidence of stroke (1.1% vs 0.8%; adjusted HR 2.16 for CABG vs MV-PCI, 95% CI 1.16 to 4.04; p=0.015) (online supplemental figure S2). No difference in all-cause mortality was observed.

## Discussion

In this nationwide retrospective cohort study, clinically significant post-procedural AF, defined by initiation and persistence of OAC therapy, was nearly 10-fold more common after CABG than after MV-PCI, despite PCI patients being older with more comorbidities. Differences in clinical outcomes at 90 days were modest: myocardial infarction was more frequent after MV-PCI, whereas stroke was more frequent after CABG, with no significant differences in all-cause mortality or major bleeding.

To capture clinically significant AF rather than brief or subclinical postoperative episodes, we used a novel, pragmatic, treatment-based proxy for post-procedural AF: OAC dispensation within 3 months post-procedure with continued use at 9 months. This approach parallels strategies in other fields where treatment serves as a surrogate for disease presence, such as antidiabetic therapy for type 2 diabetes,^19^ antidepressant use for depression^20^ and chemotherapy dispensing to identify cancer recurrence.^21^ By requiring both initiation and persistence of OAC therapy, we aimed to identify clinically relevant AF in large datasets while minimising overestimation from brief, likely insignificant episodes.

In our cohort, clinically significant post-procedural AF after MV-PCI was notably low (0.6%), consistent with the low rates generally reported after elective PCI in contemporary settings.^17^ This incidence is markedly lower than the 4–7% reported in ST-elevation myocardial infarction cohorts undergoing primary PCI, where new-onset AF often reflects transient peri-infarction arrhythmias.^12^,^16^ The low incidence in our MV-PCI cohort likely reflects the predominantly elective setting and our conservative, treatment-based definition, which captures AF episodes that are clinically recognised and lead to sustained OAC therapy in routine care. This incidence was substantially lower than after CABG and may, in part, reflect differences in procedural stress and inflammatory response between revascularisation strategies.

The reported incidence of post-procedural AF after CABG varies widely, up to 40% in some series, depending on monitoring intensity and definitions. Many such estimates likely include transient or subclinical episodes that may not recur beyond the early postoperative period. In a recent study with long-term continuous rhythm monitoring after CABG, the cumulative incidence of new-onset AF within the first year was high, but AF burden beyond 30 days was very low, highlighting uncertainty regarding which postoperative episodes warrant sustained anticoagulation.^22^ Our approach complements this literature by estimating the incidence of CABG patients who remain on long-term OAC after the procedure, thereby preferentially capturing AF cases for which clinicians have deemed sustained treatment necessary.

Although the incidence of post-procedural AF after CABG was substantially higher, the absolute difference in 90-day stroke rate was modest (0.6% vs 0.3%), indicating that multiple factors likely contribute to the elevated stroke risk observed after CABG.^23 24^ These findings are consistent with those from the SYNTAX and FREEDOM trials, which reported higher stroke rates after CABG than PCI, although neither trial reported post-procedural AF or OAC use.^25 26^ Our data provide additional insight by describing real-world patterns of OAC-treated post-procedural AF and associated outcomes in routine practice.

Conversely, MV-PCI was associated with a higher incidence of myocardial infarction, likely reflecting stent-related events or progression of non-revascularised disease. These findings are in line with trials such as EXCEL and BEST, which demonstrated increased early myocardial infarction risk after PCI in patients with complex multivessel or left main disease.^17 27^ The absence of significant differences in mortality or major bleeding highlights the multifactorial nature of post-revascularisation risk and underscores the importance of personalised secondary prevention strategies.

Subgroup analyses showed that the higher incidence of post-procedural AF after CABG versus MV-PCI was generally consistent across predefined subgroups. A significant sex interaction was observed, with AF being more frequent in males, while other subgroups exhibited similar relative patterns between the procedures. This interaction should be interpreted cautiously given multiple subgroup testing.

Our sensitivity analyses suggest that procedural complexity may influence post-procedural AF risk within the MV-PCI cohort. Using two-vessel disease as the reference, both three-vessel PCI and left main involvement showed numerically higher post-procedural AF incidence, though power was limited. While complex PCI is a known independent risk factor for adverse clinical outcomes,^28 29^ evidence linking procedural complexity specifically to post-PCI AF remains limited and warrants further research.

### Limitations

Despite the strengths of national, high-quality registries and extensive adjustment, residual confounding cannot be excluded. Treatment selection bias may exist, as patients undergoing MV-PCI and CABG may differ in ways not fully captured by registry data, such as frailty, anatomical complexity and physician preference. Additionally, using dispensed OAC prescriptions as a proxy for post-procedural AF may misclassify cases when OAC is initiated for other indications (eg, venous thromboembolism, left ventricular thrombus or arterial thromboembolism), and the absence of systematic rhythm-monitoring data prevents validation of the underlying arrhythmia or assessment of differential detection between procedures. Conversely, our approach likely underestimates the true incidence of post-procedural AF when OAC is not initiated or withheld, such as brief or asymptomatic episodes and situations where anticoagulation is avoided because of bleeding risk or frailty. Finally, differential detection and treatment thresholds between CABG and MV-PCI cannot be excluded. We required OAC persistence at 9 months to reduce transient peri-procedural treatment and to focus on sustained, clinically actionable cases.

## Conclusions

In this contemporary nationwide study, clinically significant post-procedural AF, defined by initiation and persistence of OAC therapy, was nearly 10-fold more common after CABG than after MV-PCI. Differences in clinical outcomes were modest and should be interpreted cautiously. Dispensed prescription data provide a pragmatic, scalable approach to identifying clinically actionable post-procedural AF in large datasets, but require careful interpretation and validation.

## Supplementary material

10.1136/openhrt-2026-004030 online supplemental file 1

10.1136/openhrt-2026-004030 online supplemental file 2

## Data Availability

Data may be obtained from a third party and are not publicly available.
