# Supplementary material for: Incidence of post-procedural atrial fibrillation after multivessel percutaneous coronary intervention versus coronary artery bypass grafting: a nationwide observational study
Source: Open Heart. 2026 Jul 13;13(2):e004030. doi: 10.1136/openhrt-2026-004030 (PMC13365786; doi:10.1136/openhrt-2026-004030)
Supplement: online supplemental file 1 [file openhrt-13-2-s001.pdf]

Supplementary Material

Table S1.
Figures S1-S2.

Table S1. Outcome Definitions

Outcome	Source	Definition
Dispensed prescription of oral anticoagulants	Swedish National Prescribed Drug Register	At least one dispensed prescription of an OAC (ATC codes: B01AF01, B01AF02, B01AF03, B01AE07, or B01AA03) within 90 days following revascularization, and another dispensation between days 270-450.
Stroke	National Patient Register	Ischemic stroke ICD-10: I63.0–I63.6.
Bleeding event	National Patient Register	Hemorrhagic stroke:<br>ICD-10: I60-I62.<br><br>Gastrointestinal bleeding:<br>ICD-10: K22.6, K25.0, K25.2, K25.4, K25.6, K26.0, K26.2, K26.4, K26.6, K27.0, K27.2, K27.4, K27.6, K28.0, K28.2, K28.4, K28.6, K29.0, K62.5, K92.0, K92.1, K92.2, I85.0.<br><br>Anemia-related bleeding:<br>ICD-10: D62.9, D50.0.<br><br>Other bleeding:<br>ICD-10: N42.1, N93.8, N93.9, N95.0, R04.1, R04.2, R04.8, R04.9, R21.0, R31.9, T81.0, N50.1.
Myocardial infarction	SCAAR registry	Admission for coronary angiography and/or PCI according to the Fourth Universal Definition of Myocardial Infarction (ICD-10: I21–I22).
All-cause mortality	National Population Register	All-cause mortality.

ATC = Anatomical Therapeutic Chemical classification; ICD-10 = International Classification of Diseases, 10th Revision; OAC = oral anticoagulant; SCAAR = Swedish Coronary Angiography and Angioplasty Registry.

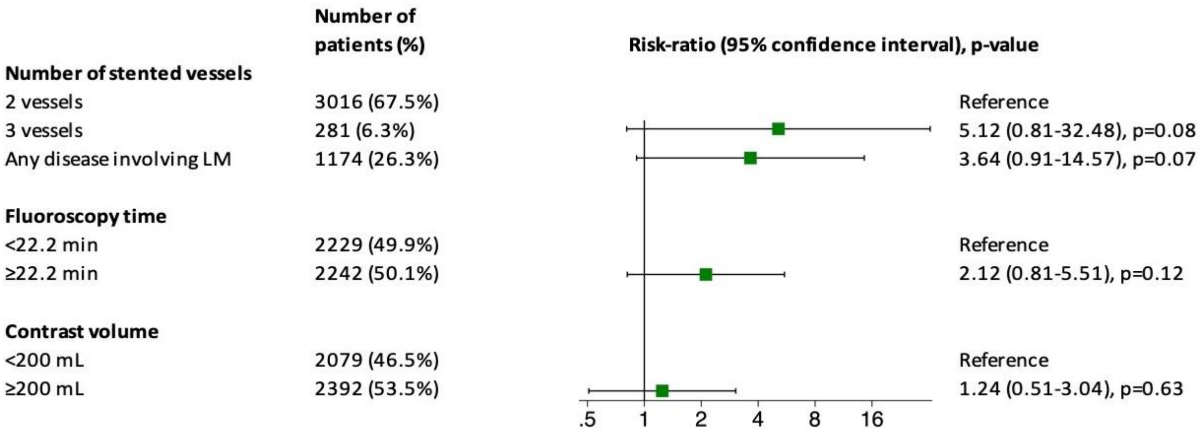

Figure S1. **Sensitivity analysis of procedural factors.** Forest plot showing adjusted risk ratios for clinically significant post-procedural atrial fibrillation associated with multivessel percutaneous coronary intervention procedural factors: number of stented vessels, fluoroscopy time, and contrast volume. LM = left main. Left main involvement may overlap with vessel-category definitions.

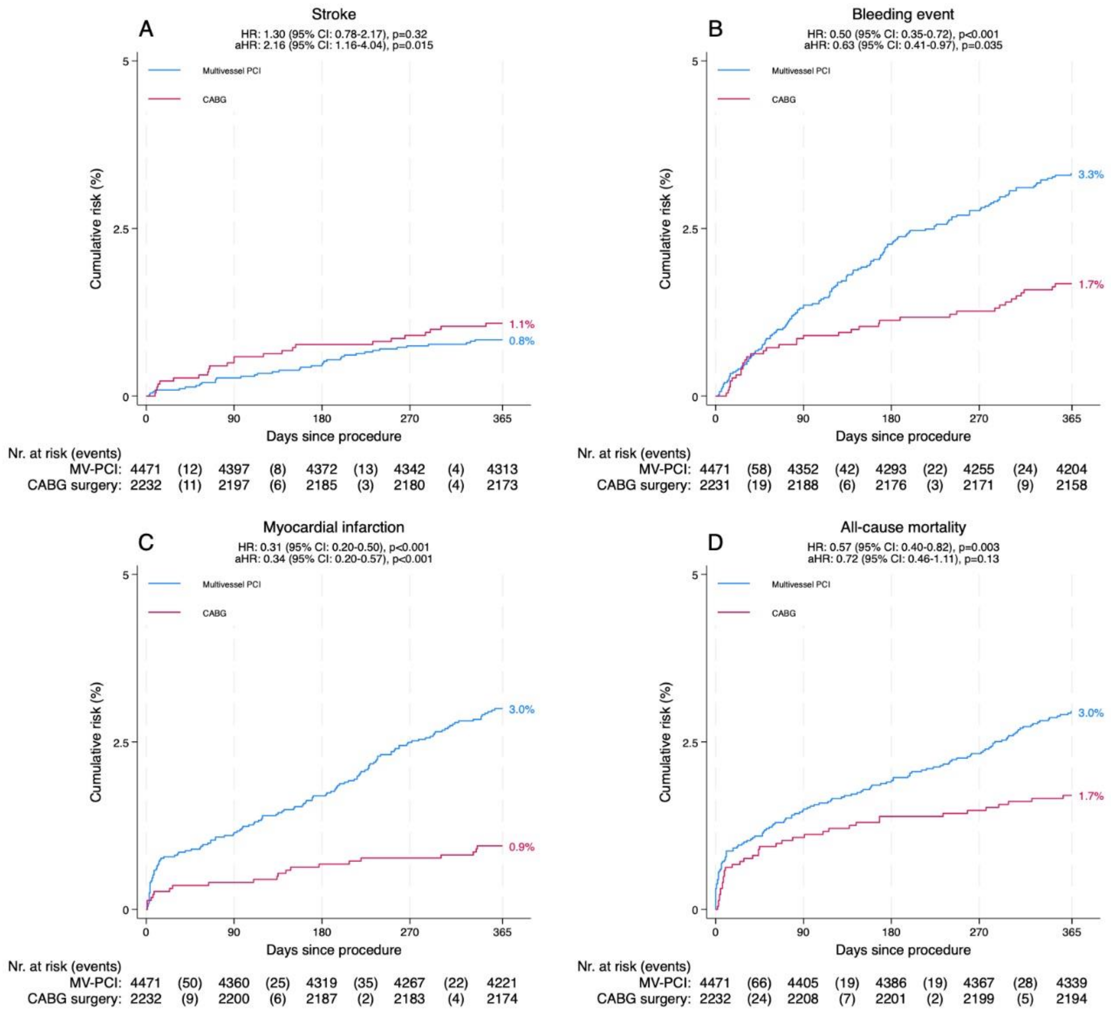

Figure S2. **One-year secondary outcomes: MV-PCI vs. CABG.** Kaplan-Meier curves illustrating the one-year event rates for (A) stroke, (B) bleeding events, (C) myocardial infarction, and (D) all-cause mortality in MV-PCI and CABG patients. Outcomes were analyzed using Kaplan-Meier estimates and both univariable and multivariable Cox proportional hazards regression. The multivariable model was adjusted for inclusion year, age, sex, diabetes mellitus, hypertension, hyperlipidemia, prior major bleeding, heart failure, chronic kidney disease, angiographic findings, smoking status, Canadian Cardiovascular Society score, and history of myocardial infarction, stroke, PCI, and CABG.

aHR = adjusted hazard ratio; CABG = coronary artery bypass grafting; HR = hazard ratio; MV-PCI = multivessel percutaneous coronary intervention.
