# Supplementary figures and images for: Incidence of post-procedural atrial fibrillation after multivessel percutaneous coronary intervention versus coronary artery bypass grafting: a nationwide observational study
Source: Open Heart. 2026 Jul 13;13(2):e004030. doi: 10.1136/openhrt-2026-004030 (PMC13365786; doi:10.1136/openhrt-2026-004030)

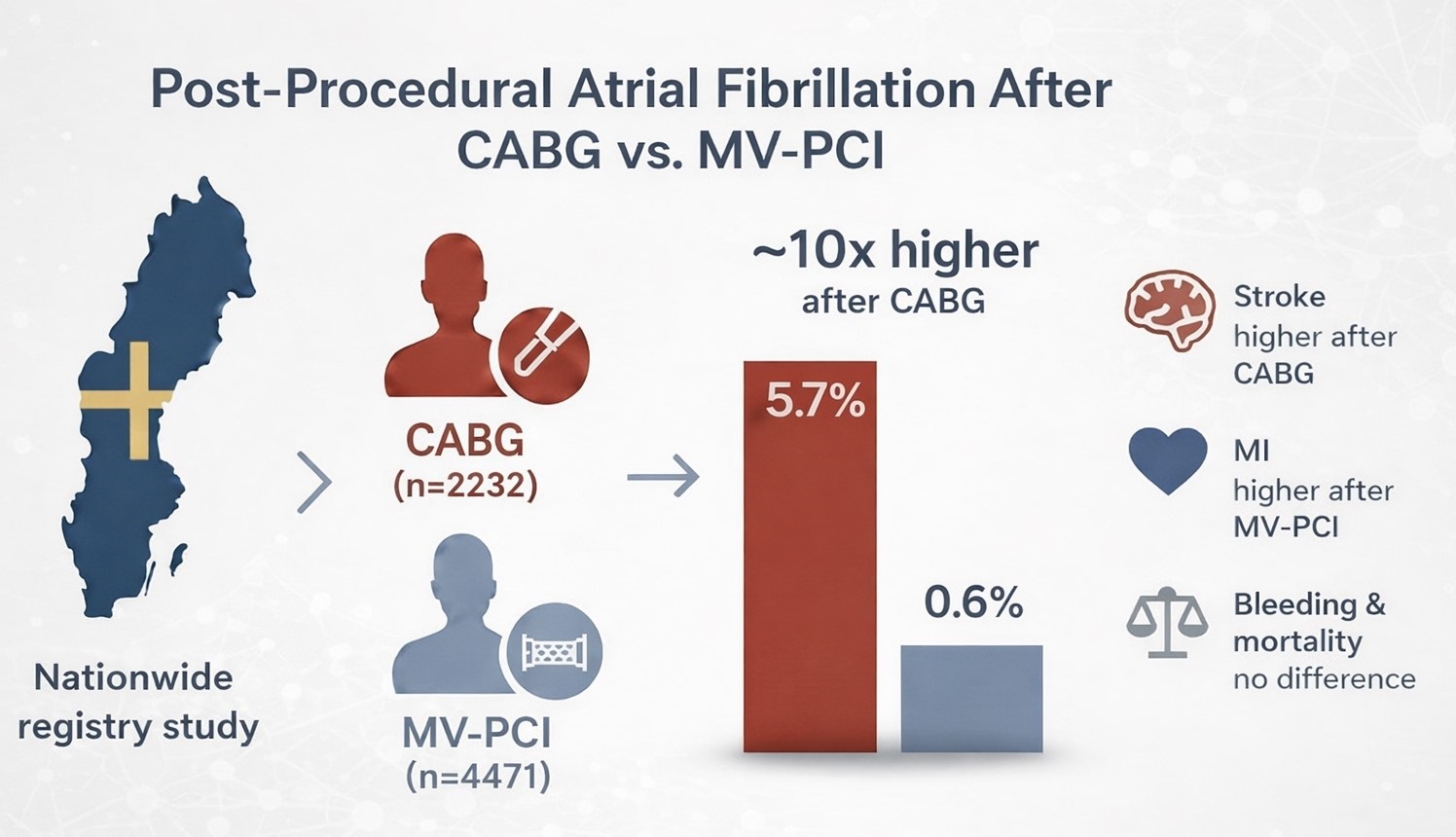

Supplement: online supplemental file 2 [file openhrt-13-2-s002.jpg]
